# Optimization of Sentinel Lymph Node Imaging Methodology Using Anionic Liposome and Hyaluronidase

**DOI:** 10.3390/pharmaceutics13091462

**Published:** 2021-09-14

**Authors:** Yu Sakurai, Miho Suzuoki, Masaki Gomi, Hiroki Tanaka, Hidetaka Akita

**Affiliations:** Laboratory of DDS Design and Drug Disposition, Graduate School of Pharmaceutical Sciences, Chiba University, 1-8-1 Inohana, Chuo-ku, Chiba City 260-0856, Japan; suzuoki@chiba-u.jp (M.S.); aema1696@chiba-u.jp (M.G.); hiroki_tanaka8922@chiba-u.jp (H.T.)

**Keywords:** liposome, sentinel lymph node, phosphatidylserine

## Abstract

The sentinel lymph node (SLN) is the first lymph node into which lymphatic fluid from tumor tissues flows. The development of a highly sensitive probe for detecting SLNs is desired for the lymph node dissection through intraoperative biopsy. We have previously shown that anionic liposomes tend to accumulate in lymph nodes and that macrophage uptake of liposomes contributes to their accumulation. In the present study, we found that among anionic lipids, phosphatidylserine (PS)-containing liposomes were substantially taken up by macrophages. We identified a new lipid composition to improve the SNL-selectivity of liposome accumulation based on Design-of-Experiment. The optimized PS-containing particles were more selectively accumulate to SLN lymph nodes than existing imaging agents indocyanine green. These results indicate the effectiveness of PS-containing anionic particles in SLN imaging.

## 1. Introduction

Lymphadenectomy is performed on cancer patients with suspected lymphatic metastasis in order to prevent postoperative recurrence [1]. In the past decades, patients with suspected lymph node (LN) metastasis were basically treated with the removal of all LNs around the tumor tissue [2]. However, the removal of lymph nodes causes lymphedema which could cause a significant decrease in the quality of life of patients. To solve this problem, lymph node metastasis is currently inspected by intraoperative observation to minimize the unnecessary dissection of lymph nodes [3]. The sentinel lymph node (SLN) is the first lymph node to receive lymphatic fluid from tumor tissue. Since the presence of metastasis in SLNs is one of the key criteria of lymphatic metastasis, a convenient method and/or probe for the intraoperative detection of SLN is important [4,5]. Intraoperative biopsy of SLN is performed especially in case of breast cancer, in that metastasize to LNs is observed in high frequency [6,7]. However, since lymphatic vessels are colorless and transparent, it is difficult to visually identify SLN connected to lymphatic vessels. Therefore, radioisotopes and fluorescent dyes such as indocyanine green (ICG) have been used as probes for intraoperative biopsy [8,9]. However, low molecular weight compounds are generally rapidly diffused from the site of administration and also, other LN, as well as SLN are stained [10].

To develop the probes that accumulate more selectively in the LN where the probes first reach (referred to as primary LN), imaging techniques using nanoparticles such as micelles and inorganic molecules are being developed [11,12,13]. We also reported on the lymphatic flow modified (LFM) mouse model that confers the non-invasive analysis of LN-to-LN kinetics of macromolecules for a long time [14]. Furthermore, using this LFM model, we comprehensively analyzed the effect of physical properties (size, surface potential) of liposomes on their kinetics in the lymphatic system. As a result, we found that anionic liposomes with cholesteryl hemisuccinate (CHEMS) with 130 nm diameter well accumulated in the primary LNs, and retained in it [15]. This property was applicable to the imaging of the sentinel lymph nodes using tumor-bearing mice. Based on the hypothesis that intra-tumoral extracellular matrix (ECM) hampers the drainage of nanoparticles into the lymphatic system, hyaluronan-degrading enzymes (HAase) were co-injected to improve the SLNs with great sensitivity. We also revealed that the accumulation of SLNs in macrophages determined their retention in lymph nodes. There are three types of macrophages in LN: subcapsular sinus macrophage (SSM), marginal sinus macrophage (MSM), and marginal cord macrophage (MCM). In the previous report, we have also shown that uptake especially by MSM and SSM of these three types of macrophages is important for the retention in LNs [15].

Based on the assumption that interaction of liposomes to CD169 macrophages is a determinant factor for LN accumulation, the purpose of this study is to find a composition that strongly binds to macrophages in LNs and hence allows the sensitive imaging of SLN using a design of experiment (DoE) method. DoE is an approach to identify the optimal conditions in a smaller number of trials by systematically constructing a group of experiments in which various conditions are organized into combinations determined based on an orthogonal array prior to experiments [16,17,18]. Preliminary results showed that 1,2-dioleoyl-sn-glycerophosphoserine (PS) has a higher affinity to the macrophages than the previously optimized CHEMS liposomes when they were incubated with cell suspensions. Therefore, PS was incorporated into one factor of DoE. A five-level, two-factor DoE was performed, including the amount of PEG modification, filter diameter for particle preparation, presence of hyaluronidase, and type of phospholipids and cholesterols. As a result, we found a new particle composition containing PS with high delivery to the sentinel lymph node. Furthermore, the imaging with this liposome was superior to that with the existing imaging agent ICG in terms of tumor selectivity and tumor retention.

## 2. Materials and Methods

### 2.1. Materials

Egg phosphatidylcholine (EPC), 1,2-dioleoyl-*sn*-glycerophosphocholine (PC) and poly(ethylene glycol) (average molecular weight is 2000) dimyristoyl-*rac*-glycerol (PEG-DMG) were purchased from NOF Corporation (Tokyo, Japan). CHEMS and PS were obtained from Avanti Polar Lipids (Alabaster, AL, USA). Cholesterol (chol) and RPMI-1640 were obtained from Merck (Burlington, MA, USA). Chloroform, 99.5% ethanol and Dulbecco’s phosphate-buffered saline without Mg^2+^ and Ca^2+^ (PBS; KCl 200 mg/L, NaCl 8000 mg/L, KH_2_PO_4_ 200 mg/L and Na_2_HPO_4_ (anhydrous) 1150 mg/L), 2-[4-(2-hydroxyethyl)piperazin-1-yl]ethanesulfonic acid (HEPES) were purchased from Nacalai tesque (Kyoto, Japan). Isoflurane was obtained from Pfizer (New York, NY, USA). Fluorescent dyes, 1,1′-dioctadecyl-3,3,3′,3′-tetramethylindodicarbocyanine (DiD) and 1,1′-dioctadecyltetramethyl indotricarbocyanine iodide (DiR) was purchased from ThermoFisher Scientific (Waltham, MA, USA). Matrigel Matrix was obtained from Becton Dickinson (Franklin Lakes, NZ, USA).

### 2.2. Liposome Preparation

All lipids were dissolved in ethanol at a concentration of 10 mM. Lipids (EPC/cholesterol derivatives = 50/50, with 1 or 5 mol% of PEG-DMG) were mixed in a glass tube, and then the solvent was evaporated in vacuo. In the DoE approach, the lipid composition was fixed to EPC/phospholipid (PC or PS)/cholesterol (chol or CHEMS) = 20/30/50. The obtained lipid thin layer was re-dissolved in 300 μL of chloroform and gently mixed with a vortex mixer. The solvent was dried by nitrogen gas. The suspension was obtained by gentle mixing 30 min after the lipid thin layer was hydrated with PBS. The suspension was passed through a polycarbonate filter with 100 and 50-nm pores several times. The size distribution of liposomes was determined with a ZetaSizer nano ZS (Malvern Panalytical, Malvern, UK). For evaluating the diameter, liposomes in PBS were analyzed by 10 repeated runs at 25 °C. For measuring zeta-potential, liposomes suspended in 10 mM HEPES buffer (pH 7.4) were analyzed by 20 repeated runs at 25 °C. To label liposomes with fluorescence, DiD or DiR was added to the lipid mixture at 0.1 mol% (for DiD) or 0.2 mol% (for DiR) of total lipids.

### 2.3. Liposomes Uptake by Macrophages Suspended from LN

Mice were sacrificed, and then inguinal LN and axillary LN were collected. The collected LN was incubated in collagenase mixture (0.1 mg/mL Collagenase IV, 0.2 mg/mL Collagenase D, 0.1 mg/mL DNase I, 1 *v*/*v*% fetal bovine serum in PRMI-1640) for 30 min at 37 °C. Cells were then washed by FACS buffer (0.5% fetal bovine serum, 0.01% sodium azide in PBS) twice. Aliquots of cells (2.0 × 10^5^ cells) were incubated with fluorescent liposomes at 50 μM for 2 h at 37 °C. After cells were washed by FACS buffer twice, cells were incubated with 10 μg/mL CD16/32 antibody (Biolegend #101302) in 50 μL FACS buffer for 30 min on ice for blocking Fc receptor CD16 and CD32. After washed, cells were stained by Brilliant Violet 421 anti-mouse/human CD11b (Biolegend #101236), FITC anti-mouse F4/80 (Biolegend #123108), PE-Dazzle594 anti-mouse CD169 (Biolegend #142424) for 30 min on ice, tapped every 10 min. The cell suspension was then incubated with 0.5 μg/mL 7-Aminoactinomycin D (7-AAD, Biolegend #420404) for 5 min to stain dying cells after twice washing, and then cell suspensions were analyzed with a flow cytometer Novocyte (Agilent Technologies, Santa Clara, CA, USA). We regarded CD11b^high^/F4/80^−^/CD169^+^ cells, CD11b^high^/F4/80^+^/CD169^+^ and CD11b^high^/F4/80^+^/CD169^−^ cells as SSM, MSM and MCM, respectively.

### 2.4. Cell Culture

Murine breast cancer 4T1 cells were obtained from ATCC. 4T1 cells were maintained in RPMI-1640 supplemented with 10% fetal bovine serum, 100 U/mL penicillin, 100 μg/mL streptomycin under 5% CO_2_ humidified air at 37 °C.

### 2.5. Animal Model

To establish an orthotopic breast cancer model, a mixture with 50 μL of Matrigel Matrix and 4T1 cells (5 × 10^5^ cells in 50 μL PBS) was injected into the mammary fat bad of BALB/c mice (5 or 6-weeks-old, female) under isoflurane anesthesia. When tumor volume reached Day 18, tumor-bearing mice were used for SLN imaging. The LN in the inguinal meridian closest to the transplant site was considered the SLN. HAase (2500 U) was administered into tumor tissue, whose amount was determined based on our and other groups’ previous reports [15,19,20]. The experimental protocols were reviewed and approved by the Chiba University Animal Care Committee in accordance with the Guide for Care and Use of Laboratory Animals (Dou3-253).

### 2.6. SLN Imaging

To detect SLN, 0.1 mol% DiR-labeled liposome (250 nmol) was co-injected in breast tumor with 2500 U HAase in PBS. In comparison, ICG (125 μg/50 μL) was intratumorally administered. Mice were sacrificed 6 h after the injection and then observed with an IVIS Lumina II (Waltham, MA, PerkinElmer). Tumor tissues were excised prior to the detection to avoid saturation of fluorescent intensity because most liposomes remained in tumor tissues. SLN selectivity was calculated by the below equation:

SLN selectivity = fluorescence in SLN/fluorescence in axillary LN

### 2.7. DoE Approach

In the DoE approach, the main factors are assigned based on L_16_(2^15^)-type orthogonal array based on Taguchi’s method [21]. All of the main factors have two levels as shown in Table 1. The animal experiment was performed using two mice per condition. To estimate the effect of each factor on SLN selectivity, an analysis of variance was carried out. The variance ratios (*F*) between each factor and the residual were analyzed by *F*-test. The optimum condition was estimated by the total value of the main factors and the interactions.

### 2.8. Statistical Analysis

For pair-wise comparison, Student’s *t*-test was performed. For multiple comparison, Tukey’s range test was performed. Both comparisons were carried out with a GraphPad Prism 9.1.2 software (GraphPad Software). *p*-value < 0.05 was regarded as a statistically significant difference.

## 3. Results

### 3.1. Affinity of PS-Containing Liposomes to LN-Residential Macrophages

**Figure 1:**
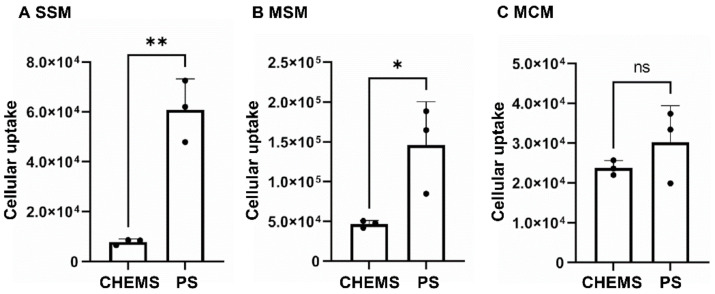
Uptake of anionic liposomes with CHEMS or PS by macrophages. Cell suspension was isolated from mice LNs and then incubated with each DiD-labeled liposome at 50 μM for 2 h. After washing free liposomes, DiD fluorescence in (**A**) SSM, (**B**) MSM, and (**C**) MCM were analyzed by flow cytometry (*n* = 3). *: *p* < 0.05, **: *p* < 0.01, ns: not statistically significant.

PS is reported to have a high affinity to macrophages via the T cell immunoglobulin and mucin domain-containing protein 4 (Tim4) [22,23]. As already mentioned, in previous reports we showed that liposome uptake by macrophages is a crucial factor for the retention of liposomes in LN [15]. Therefore, we hypothesized that PS may have a higher affinity for macrophages among anionic lipids, and hence increased accumulation to LN. To test this hypothesis, we first evaluated the uptake of PS-containing liposomes to the macrophages in ex vivo cell suspensions isolated from LN. The typical population was 31.4% (SSM), 11.9% (MSM) and 37.5% (MCM), which is consistent with the previous report [24]. The uptakes of PS-containing liposomes to three types of macrophages were compared with CHEMS-containing ones by flow cytometry. The results showed that the uptakes of PS-containing liposomes increased 7.8-fold and 3.1-fold in SSM and MSM, respectively, compared to CHEMS liposomes (Figure 1). On the other hand, the uptake of these two types of liposomes was comparable.

### 3.2. Optimization of SLN Imaging Using Anionic Liposomes

To optimize the lipid composition for SLN-selective imaging, 16 runs were performed for DoE (Table 1). The characterization of liposomes and the observed results are listed in Table 2. The diameter of liposomes ranged from 76 to 138 nm, zeta-potential from 0 to −33 mV. Polydisepersity Index (*PDI*) of all samples below 0.11, indicating the monodispersity of samples. After these liposomes were intratumorally administered, the selectivity of each liposome in SLN was evaluated.

**Figure 2:**
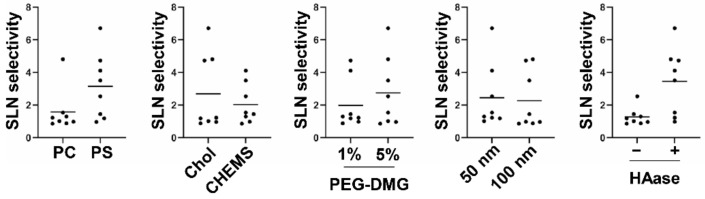
The effect of each main factor (Phospholipid, Cholesterol, PEG amount, Pore size, HAase treatment) on SLN selectivity. The number of each data set was 8. Circles and bars indicate each data point and mean.

To evaluate the effect of each main factor and the interaction among them on SLN selectivity, analysis of variance was performed, and the *p*-value was calculated by F-test (Table 3). The interactions by a combination of the main factors (X1 × 2, X1 × 3, X3 × 4, X4 × 5) were regarded as a residual due to low variance. The entire data set is shown in Table S1. As a consequence, the main factors X1 (phospholipid: PS), X2 (cholesterol: chol), X3 (PEG: 5%), X5 (HAase: +) were statistically significant. For vitalization, all of the data points in a group of each indicated main factor were plotted (Figure 2). Further, the interactions X1 × 4, X1 × 5, X2 × 5 were also statistically significant. The estimated optimum condition was X1 (PS), X2 (Chol), X3 (5%), X4 (filter pore size 50 nm), and X5 HAase (+), whose SLN selectivity was calculated as 6.457 (95% confidence interval (CI): 5.567~7.346).

### 3.3. Verification Study to the Result of DoE

**Figure 3:**
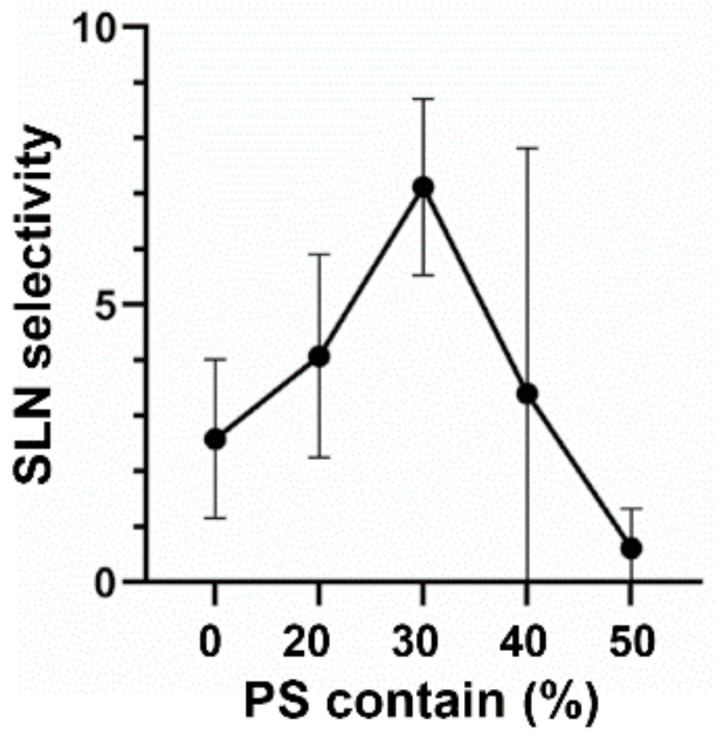
The optimization of PS ratio. The liposomes with lipid compositions of EPC/PS/Chol (50-X/X/50, moral ratio) were intratumorally administered into tumor-bearing mice. SLN selectivity was determined by calculating SLN accumulation/axillary LN accumulation. Data represent mean ± standard deviation (*n* = 3).

To further optimize the PS ratio, various amounts of PS (20–50%, molar ratio) were incorporated into lipid composition instead of EPC (Figure 3). As a result, the highest SLN selectivity was achieved in 30% PS-containing liposomes, in which a 2.8-fold higher selectivity was obtained compared with PS-free liposomes. This optimum PS content (30%) is the same lipid composition that was determined by the DoE approach described above.

**Figure 4:**
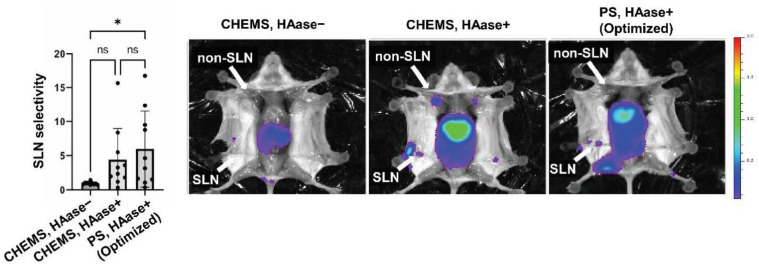
SLN imaging by the optimized liposomes. Mice were treated with liposomes in the presence or absence of HAase, and then imaged with an IVIS imaging system. Inguinal LN and axillary LN were considered SLN and non-SLN, respectively. *: *p* < 0.05, ns: not statistically significant (*n* = 10).

To verify the significance of the optimized liposome formulation, we then compared the optimized condition to others (Figure 4). When CHEMS was included as an anionic lipid, SLN selectivity was 0.961 in the absence of HAase and 4.402 in the presence of HAase. These values were lower than that of PS-containing liposomes with HAase, while not statistically different. In the case of CHEMS, HAase+ group, other LNs except SLN were also detected. As CHEMS liposomes would associate less with LN macrophages, CHEMS may be drained to the afferent efferent lymphatic vessel towards secondary LNs. On the other hand, PS-containing liposomes were detected only in SLN, suggesting that PS-containing liposomes could retain in LNs due to strong binding to macrophages. SLN selectivity of the optimized liposome is within an estimated 95% CI (5.567~7.346). A large variance was observed in the experiment. This is probably due to large individual differences in the formation of lymphatic vessels that pass through SLNs when they are transferred to liposomes.

### 3.4. Comparative Study of Optimized Methodology with ICG

**Figure 5:**
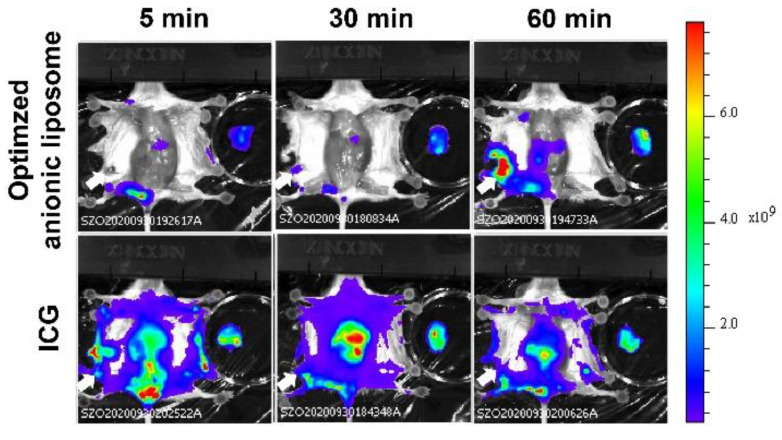
Comparison of SLN selectivity between liposomal imaging and ICG. Mice were imaged 5, 30 and 60 min after the intratumoral injection of each imaging agents. Independent two individuals were observed in each condition.

To evaluate whether the optimized liposomes are suitable for SLN imaging compared to the existing imaging agents ICG, the whole body was imaged by IVIS^@^ after the intra-tumoral injection of optimized PS-containing liposome, or ICG (Figure 5). As ICG diffused through the whole of the body, even 5 min after the treatment, SLN was hardly identified. Additionally, LN in the opposite site was further stained at 1 h after the injection. On the other hand, the optimized liposomes were detected only in the limited loci: the injection site and SLN. The accumulation of liposomes in the liver may be accounted for by assuming that a part of the liposomes was leaked to the blood circulation.

## 4. Discussion

In this study, we focused on the ability of PS to be recognized by macrophages in LN. To obtain the suitable formulation, we performed DoE Analysis with five factors: phospholipids, cholesterols, filter diameter, HAase, PEG amount. As a consequence, we revealed that PS/EPC/Chol (30/20/50, molar ratio) with 5 mol% PEG-lipid in the presence of HAase is the best treatment for imaging SLN. This condition is superior to existing imaging reagent ICG in terms of selectivity.

For the efficient accumulation and retention of liposomes in SLN two processes must be optimized; (1) drainage from tumor tissue to the lymphatic system, and (2) retention in the first lymph node reached. In this development process, we applied the DoE to efficiently identify the adequate combination of factors concerning the lipid composition, size, and adjuvant. As a result, the employment of PS is one of the key factors for selective SLN imaging. Considering PEG amount, 5 mol% modification was superior to 1 mol% modification. This seems to be inconsistent with the assumption that macrophage uptake is important for LN accumulation. After the intratumoral injection of liposomes, many types of cells such as cancer cells and tumor-associated macrophages could capture liposomes, which would cause a decrease in the entrance of liposomes into the lymphatic system. PEGylation would allow liposomes to circumvent the unnecessary uptake in the tumor tissue. Therefore, highly PEGylation would demonstrate better SLN imaging capacity.

It is known that Tim4, which is expressed on macrophages, is reported to recognize both the acyl group and head group in the PS molecule [22]. PS-mediated uptake is also involved with endogenous particles by macrophages [25,26]. In particular, the uptake of these vesicles to SSM and MSM in LNs plays a key role in immune suppression or tolerance [27], whereas the role of MCM has not been reported yet [28]. However, another report revealed that allograft-derived extracellular vesicles enhanced the immunity in the donor, and then consequently induced the rejection of allograft [29]. The precise role of this PS-mediated uptake is currently controversial. However, in many cases, PS-containing liposomes were expected to be taken up by SSM and MSM, while the mechanism for the comparable uptake between CHEMS- and PS-containing liposomes is still not clear (Figure 1). In addition, the uptake of these liposomes to MCM was lower than that of other macrophages, suggesting that the intrinsic phagocytic capacity in MCM is less than sinus macrophages. Thus, it is plausible that the contribution of MCM uptake to the overall retention of LNs might be poor. Our previous reports and non-published data have shown that the incorporation of negatively charged lipids (i.e., phosphatidic acid and phosphatidylglycerol), was not superior to that of LN accumulation of CHEMS liposomes. This suggests that physicochemical property (anionic charge) cannot simply explain the extensive uptake of PS-containing liposomes to the macrophages [15]. We speculate that PS liposomes are taken up by macrophages and lodged in lymph nodes using not only the affinity of the anionic nature itself but also the affinity of the PS receptor.

In the process of the DoE, a statistically significant interaction was found in the following combinations: Phospholipid x HAase, Phospholipid x Diameter of Filter, and Cholesterol x HAase (Table 3). In the previous study, we demonstrated that collagen removal affected the LN accumulation less [15]. This result indicated that hyaluronan is the more decisive factor among ECM for the prevention of nanoparticle diffusion. In this study, SLN-selective imaging was succeeded by PS-containing liposomes with the aid of co-injection of HAase (Figure 2), which is under clinical trial for the cure of pancreatic cancer with a combination of anti-cancer reagents [30]. This suggests that the ECM including hyaluronan in tumor tissue is also a major obstacle to the drainage of PS-containing liposomes. The mechanism for the significant interaction between the cholesterol content and co-injection of HAase is not clear. It is possible that the cholesterol content affected the stability of the liposome when the drainage of liposomes was accelerated by the co-injection of HAase.

Finally, as to the size of liposomes, it is well-accepted that small-sized molecules (<10 nm) enter the blood circulation after the subcutaneous administration, while the large-sized molecules (>100 nm) preferably enter the lymphatic system [31]. This is generally explained by the morphological difference of blood vessel and lymphatic vessel: the blood endothelial cells are tightly adherent to each other through the interaction of membrane proteins [32], whereas the junction of lymphatic endothelial cells is looser than blood endothelial cells [33]. As shown in Figure 2, the size (i.e., the diameter of the filter) per se was not a main factor for the SLN-selective accumulation even when the diameter was varied from approximately 80–85 nm (filtration through smaller pore) to 100–140 nm (filtration with larger pore). However, DoE approach revealed that the effect of size appealed when PS-containing liposome was used. The mechanism for this phenomenon was not clear. However, it is possible that the advantage of the smaller size become obvious under the situation that the electrostatic repulsion between the liposome and extracellular matrix can be a driving force for the drainage of liposomes.

## 5. Conclusions

In this study, we identified the optimum condition for detecting SLN using the DoE approach. As a consequence, the PS-containing liposome is a promising probe for the SLN-selective detection when they were combined with HAase since it satisfies the efficient accumulation and retention in SLN. PS-containing liposomes are internalized into CD169-positive macrophages, which could contribute to LN accumulation. In addition, PS-containing liposomes are superior to the currently available reagent, ICG for SLN imaging. The particle might be suitable for antigen/adjuvant delivery for cancer immune therapy since the accumulation in macrophages is a driving force for the extensive accumulation in the lymph node.

## Figures and Tables

**Table 1:** Experimental matrix of DofE.

Run	Phospholipid X1	Cholesterol X2	PEG Molar Ratio X3	Diameter of Filter X4	HAase X5
1	PC	Chol	1%	50	+
2	PC	Chol	1%	100	−
3	PC	Chol	5%	50	−
4	PC	Chol	5%	100	+
5	PC	CHEMS	1%	50	−
6	PC	CHEMS	1%	100	+
7	PC	CHEMS	5%	50	+
8	PC	CHEMS	5%	100	−
9	PS	Chol	1%	50	−
10	PS	Chol	1%	100	+
11	PS	Chol	5%	50	+
12	PS	Chol	5%	100	−
13	PS	CHEMS	1%	50	+
14	PS	CHEMS	1%	100	−
15	PS	CHEMS	5%	50	−
16	PS	CHEMS	5%	100	+

**Table 2:** Physicochemical properties and SLN selectivity of conditions.

Run	Z-Average (nm)	Zeta Potential (mV)	*PDI*	SLN Selectivity
1	84	0.5	0.036	1.221
2	119	−0.2	0.072	0.873
3	81	0.1	0.056	1.015
4	116	0.2	0.023	4.811
5	83	−28.9	0.082	1.298
6	134	−29.7	0.041	0.977
7	76	−12.9	0.074	1.534
8	138	−11.2	0.074	0.858
9	81	−31.2	0.077	1.183
10	108	−31.5	0.085	4.730
11	84	−13.7	0.075	6.707
12	115	−14.8	0.060	0.964
13	79	−31.8	0.071	4.108
14	101	−32.6	0.107	1.444
15	76	−14.7	0.081	2.536
16	118	−12.9	0.059	3.502

*PDI*: Polydispersity index.

**Table 3:** Effect of each factor against SLN selectivity (ILN to ALN ratio).

Factor	*F*	*p*-Value	Factor	*F*	*p*-Value
X1	48.2	0.002	X1 × 4	12.2	0.025
X2	8.38	0.044	X1 × 5	21.6	0.010
X3	11.3	0.028	X2 × 3	7.26	0.054
X4	0.634	0.470	X2 × 4	4.74	0.095
X5	92.3	<0.001	X2 × 5	27.2	0.007
			X3 × 5	7.44	0.053

## Data Availability

Not applicable.

## Supplementary Materials

The following are available online at https://www.mdpi.com/article/10.3390/pharmaceutics13091462/s1, Table S1: title Summary of DoE results.

## References

[1] Hsueh E.C., Hansen N., Giuliano A.E. (2000). Intraoperative lymphatic mapping and sentinel lymph node dissection in breast cancer. CA Cancer J. Clin..

[2] Giuliano A.E., Kirgan D.M., Guenther J.M., Morton D.L. (1994). Lymphatic mapping and sentinel lymphadenectomy for breast cancer. Ann. Surg..

[3] Blaheta H.J., Schittek B., Breuninger H., Garbe C. (2001). Detection of micrometastasis in sentinel lymph nodes of patients with primary cutaneous melanoma. Recent Results Cancer Res..

[4] Doepker M.P., Zager J.S. (2015). Sentinel lymph node mapping in melanoma in the twenty-first century. Surg. Oncol Clin. N. Am..

[5] Reintgen M., Kerivan L., Reintgen E., Swaninathan S., Reintgen D. (2016). Breast Lymphatic Mapping and Sentinel Lymph Node Biopsy: State of the Art: 2015. Clin. Breast Cancer.

[6] Giuliano A.E., Jones R.C., Brennan M., Statman R. (1997). Sentinel lymphadenectomy in breast cancer. J. Clin. Oncol..

[7] Giuliano A.E., Dale P.S., Turner R.R., Morton D.L., Evans S.W., Krasne D.L. (1995). Improved axillary staging of breast cancer with sentinel lymphadenectomy. Ann. Surg..

[8] Matsuura Y., Ichinose J., Nakao M., Okumura S., Mun M. (2020). Recent fluorescence imaging technology applications of indocyanine green in general thoracic surgery. Surg. Today.

[9] Goyal A. (2018). New Technologies for Sentinel Lymph Node Detection. Breast Care.

[10] Sugie T., Sawada T., Tagaya N., Kinoshita T., Yamagami K., Suwa H., Ikeda T., Yoshimura K., Niimi M., Shimizu A. (2013). Comparison of the indocyanine green fluorescence and blue dye methods in detection of sentinel lymph nodes in early-stage breast cancer. Ann. Surg. Oncol..

[11] Pan D., Cai X., Kim B., Stacy A.J., Wang L.V., Lanza G.M. (2012). Rapid synthesis of near infrared polymeric micelles for real-time sentinel lymph node imaging. Adv. Healthc. Mater..

[12] Lee S.B., Yoon G., Lee S.W., Jeong S.Y., Ahn B.C., Lim D.K., Lee J., Jeon Y.H. (2016). Combined Positron Emission Tomography and Cerenkov Luminescence Imaging of Sentinel Lymph Nodes Using PEGylated Radionuclide-Embedded Gold Nanoparticles. Small.

[13] Winter A., Engels S., Goos P., Suykers M.C., Gudenkauf S., Henke R.P., Wawroschek F. (2019). Accuracy of Magnetometer-Guided Sentinel Lymphadenectomy after Intraprostatic Injection of Superparamagnetic Iron Oxide Nanoparticles in Prostate Cancer: The SentiMag Pro II Study. Cancers.

[14] Yamaji Y., Akita S., Akita H., Miura N., Gomi M., Manabe I., Kubota Y., Mitsukawa N. (2018). Development of a mouse model for the visual and quantitative assessment of lymphatic trafficking and function by in vivo imaging. Sci. Rep..

[15] Gomi M., Sakurai Y., Okada T., Miura N., Tanaka H., Akita H. (2021). Development of Sentinel LN Imaging with a Combination of HAase Based on a Comprehensive Analysis of the Intra-lymphatic Kinetics of LPs. Mol. Ther..

[16] Terada T., Kulkarni J.A., Huynh A., Chen S., van der Meel R., Tam Y.Y.C., Cullis P.R. (2021). Characterization of Lipid Nanoparticles Containing Ionizable Cationic Lipids Using Design-of-Experiments Approach. Langmuir.

[17] Liu X., Bahloul B., Lai Kuen R., Andrieux K., Roques C., Scherman D. (2021). Cationic lipid nanoparticle production by microfluidization for siRNA delivery. Int. J. Pharm..

[18] Deshkar S.S., Bhalerao S.G., Jadhav M.S., Shirolkar S.V. (2018). Formulation and Optimization of Topical Solid Lipid Nanoparticles based Gel of Dapsone Using Design of Experiment. Pharm. Nanotechnol..

[19] Gong H., Chao Y., Xiang J., Han X., Song G., Feng L., Liu J., Yang G., Chen Q., Liu Z. (2016). Hyaluronidase To Enhance Nanoparticle-Based Photodynamic Tumor Therapy. Nano Lett..

[20] Eikenes L., Tari M., Tufto I., Bruland O.S., de Lange Davies C. (2005). Hyaluronidase induces a transcapillary pressure gradient and improves the distribution and uptake of liposomal doxorubicin (Caelyx) in human osteosarcoma xenografts. Br. J. Cancer.

[21] Kacker R.N., Lagergren E.S., Filliben J.J. (1991). Taguchi’s Orthogonal Arrays Are Classical Designs of Experiments. J. Res. Natl. Inst. Stand. Technol.

[22] Miyanishi M., Tada K., Koike M., Uchiyama Y., Kitamura T., Nagata S. (2007). Identification of Tim4 as a phosphatidylserine receptor. Nature.

[23] Tietjen G.T., Gong Z., Chen C.H., Vargas E., Crooks J.E., Cao K.D., Heffern C.T., Henderson J.M., Meron M., Lin B. (2014). Molecular mechanism for differential recognition of membrane phosphatidylserine by the immune regulatory receptor Tim4. Proc. Natl. Acad. Sci. USA.

[24] Phan T.G., Green J.A., Gray E.E., Xu Y., Cyster J.G. (2009). Immune complex relay by subcapsular sinus macrophages and noncognate B cells drives antibody affinity maturation. Nat. Immunol..

[25] Rodriguez-Manzanet R., Sanjuan M.A., Wu H.Y., Quintana F.J., Xiao S., Anderson A.C., Weiner H.L., Green D.R., Kuchroo V.K. (2010). T and B cell hyperactivity and autoimmunity associated with niche-specific defects in apoptotic body clearance in TIM-4-deficient mice. Proc. Natl. Acad. Sci. USA.

[26] Matsumoto A., Takahashi Y., Nishikawa M., Sano K., Morishita M., Charoenviriyakul C., Saji H., Takakura Y. (2017). Role of Phosphatidylserine-Derived Negative Surface Charges in the Recognition and Uptake of Intravenously Injected B16BL6-Derived Exosomes by Macrophages. J. Pharm. Sci..

[27] Pucci F., Garris C., Lai C.P., Newton A., Pfirschke C., Engblom C., Alvarez D., Sprachman M., Evavold C., Magnuson A. (2016). SCS macrophages suppress melanoma by restricting tumor-derived vesicle-B cell interactions. Science.

[28] Bordet E., Fretaud M., Crisci E., Bouguyon E., Rault S., Pezant J., Pleau A., Renson P., Giuffra E., Larcher T. (2019). Macrophage-B Cell Interactions in the Inverted Porcine Lymph Node and Their Response to Porcine Reproductive and Respiratory Syndrome Virus. Front. Immunol..

[29] Zeng F., Chen Z., Chen R., Shufesky W.J., Bandyopadhyay M., Camirand G., Oberbarnscheidt M.H., Sullivan M.L.G., Baty C.J., Yang M.Q. (2021). Graft-derived extracellular vesicles transported across subcapsular sinus macrophages elicit B cell alloimmunity after transplantation. Sci. Transl. Med..

[30] Doherty G.J., Tempero M., Corrie P.G. (2018). HALO-109-301: A Phase III trial of PEGPH20 (with gemcitabine and nab-paclitaxel) in hyaluronic acid-high stage IV pancreatic cancer. Future Oncol..

[31] Trevaskis N.L., Kaminskas L.M., Porter C.J. (2015). From sewer to saviour—targeting the lymphatic system to promote drug exposure and activity. Nat. Rev. Drug Discov..

[32] Palmeri D., van Zante A., Huang C.C., Hemmerich S., Rosen S.D. (2000). Vascular endothelial junction-associated molecule, a novel member of the immunoglobulin superfamily, is localized to intercellular boundaries of endothelial cells. J. Biol. Chem..

[33] Jalkanen S., Salmi M. (2020). Lymphatic endothelial cells of the lymph node. Nat. Rev. Immunol..

