# Supplementary material for: Optimization of Sentinel Lymph Node Imaging Methodology Using Anionic Liposome and Hyaluronidase
Source: Pharmaceutics. 2021 Sep 14;13(9):1462. doi: 10.3390/pharmaceutics13091462 (PMC8465215; doi:10.3390/pharmaceutics13091462)
Supplement: Supplementary file 1 [file pharmaceutics-13-01462-s001.zip › pharmaceutics-1365591-SM.pdf]

pharmaceutics

Supplementary Materials:

Optimization of anionic liposome with hyaluronidase for sentinel lymph node imagine

Yu Sakurai, Miho Suzuoki, Masaki Gomi, Hiroki Tanaka and Hidetaka Akita

Table S1. Summary of DoE results.

Factors	Sum of Square	Freedom	Variance	*F*-value	*P*-value	Significance
M	89.12	1	89.12			
Phospholipid X1	9.900	1	9.90	48.20	0.0023	**
Cholesterol X2	1.72	1	1.72	8.38	0.0443	*
PEG amount X3	2.32	1	2.32	11.30	0.0283	*
Filter pore size X4	0.13	1	0.13	0.63	0.4704	
HAase X5	18.96	1	18.96	92.30	0.0007	**
X2 X3	1.49	1	1.49	7.26	0.0543	
X1 X4	2.51	1	2.52	12.24	0.0249	*
X2 X4	0.97	1	0.97	4.74	0.0951	
X3 X5	1.52	1	1.53	7.44	0.0526	
X2 X5	5.58	1	5.58	27.15	0.0065	**
X1 X5	4.43	1	4.43	21.57	0.0097	**
Residual	0.82	4	0.21			
Total	139.49	16				

* $p < 0.05$; ** $p < 0.001$.
